# Incidence, course, and outcome of *Clostridium difficile* infection in children with hematological malignancies or undergoing hematopoietic stem cell transplantation

**DOI:** 10.1007/s10096-018-3316-5

**Published:** 2018-07-05

**Authors:** Małgorzata Salamonowicz, T. Ociepa, J. Frączkiewicz, A. Szmydki-Baran, M. Matysiak, K. Czyżewski, M. Wysocki, P. Gałązka, P. Zalas-Więcek, N. Irga-Jaworska, E. Drożyńska, O. Zając-Spychała, J. Wachowiak, O. Gryniewicz-Kwiatkowska, A. Czajńska-Deptuła, B. Dembowska-Bagińska, L. Chełmecka-Wiktorczyk, W. Balwierz, M. Bartnik, K. Zielezińska, T. Urasiński, R. Tomaszewska, T. Szczepański, M. Płonowski, M. Krawczuk-Rybak, F. Pierlejewski, W. Młynarski, Z. Gamrot-Pyka, M. Woszczyk, Z. Małas, W. Badowska, A. Urbanek-Dądela, G. Karolczyk, W. Stolpa, G. Sobol-Milejska, A. Zaucha-Prażmo, J. Kowalczyk, J. Goździk, E. Gorczyńska, K. Jermakow, A. Król, A. Chybicka, M. Ussowicz, K. Kałwak, J. Styczyński

**Affiliations:** 1 Department of Pediatric Stem Cell Transplantation, Hematology and Oncology, Medical University, Wroclaw, Borowska Street 213, 50-556 Wrocław, Poland; 2 Department of Pediatric Hemato-Oncology and Gastroenterology, Pomeranian Medical University, Szczecin, Poland; 3 Department of Pediatric Hematology and Oncology, Medical University, Warszawa, Warszawa, Poland; 4 Department of Pediatric Hematology and Oncology, Collegium Medicum, Nicolaus Copernicus University Torun, Bydgoszcz, Poland; 5 Department of Pediatric Surgery, Collegium Medicum, Nicolaus Copernicus University Torun, Bydgoszcz, Poland; 6 Department of Microbiology, Collegium Medicum, Nicolaus Copernicus University Torun, Bydgoszcz, Poland; 7 Department of Pediatrics, Hematology and Oncology, Medical University, Gdansk, Gdansk, Poland; 8 Department of Pediatric Oncology, Hematology and Transplantology, University of Medical Sciences, Poznan, Poland; 9 Department of Oncology, Children’s Memorial Health Institute, Warszawa, Poland; 10 Department of Pediatric Oncology and Hematology, University Children’s Hospital, Jagiellonian University Collegium Medicum, Krakow, Poland; 11 Department of Pediatric Hematology and Oncology, Silesian Medical University, Zabrze, Poland; 12 Department of Pediatric Oncology and Hematology, Medical University, Bialystok, Bialystok, Poland; 13 Department of Pediatric Oncology, Hematology and Diabetology, Medical University, Lodz, Lodz, Poland; 14 Division of Pediatric Hematology and Oncology, Chorzow Pediatric and Oncology Center, Chorzow, Poland; 15 Division of Pediatric Hematology and Oncology, Children Hospital, Olsztyn, Olsztyn, Poland; 16 Division of Pediatric Hematology and Oncology, Children Hospital, Kielce, Kielce, Poland; 17 Division of Pediatric Oncology, Hematology and Chemotherapy, Department of Pediatric, Silesian Medical University, Katowice, Poland; 18 Department of Pediatric Hematology, Oncology and Stem Cell Transplantation, Medical University, Lublin, Lublin, Poland; 19 Stem Cell Transplant Center, University Children’s Hospital, Department of Clinical Immunology and Transplantology, Jagiellonian University Collegium Medicum, Krakow, Poland; 20 Department of Microbiology, Medical University, Wroclaw, Wroclaw, Poland

**Keywords:** *Clostridium difficile*, Malignant diseases, Pediatric hematology and oncology, Hematopoietic stem cell transplantation, Children

## Abstract

*Clostridium difficile* infection (CDI) is one of the most common causes of nosocomial infectious diarrhea in children during anticancer therapy or undergoing hematopoietic stem cell transplantation (HSCT) in Europe. Immunosuppression in these patients is a risk factor for CDI. Malignant diseases, age, acute graft-versus-host disease (aGVHD), HLA mismatch, or use of total body irradiation may play an important role in CDI course. The aim of this study was to evaluate the incidence, course, and outcome of CDI in children treated for malignancy or undergoing HSCT. Between 2012 and 2015, a total number of 1846 patients were treated for malignancy in Polish pediatric oncological centers (PHO group) and 342 underwent transplantation (HSCT group). In PHO group, episodes of CDI occurred in 210 patients (14%). The incidence of CDI was higher in patients with hematological malignancies in comparison to that with solid tumors. Patients with acute myeloblastic leukemia had shorter time to episode of CDI than those with acute lymphoblastic leukemia. Patients over 5 years and treated for acute leukemia had more severe clinical course of disease in PHO group. In HSCT group, CDI occurred in 29 (8%) patients. The incidence of CDI was higher in patients transplanted for acute leukemia. The recurrence rate was 14.7% in PHO and 20.7% in HSCT patients. CDI incidence was highest in patients with hematological malignancies. Most of patients experienced mild CDI. Age < 5 years and diagnosis other than acute leukemia were the positive prognostic factors influencing clinical CDI course.

## Introduction

*Clostridium difficile* (CD) was described for the first time in 1935. It was found in stool specimens from healthy neonates and classified as a commensal [1], not being connected with disease until 1978 [2, 3]. Nowadays, *Clostridium difficile* infection (CDI) has become one of the most common causes of nosocomial infectious diarrhea in Europe and in the USA, resulting in a high morbidity and mortality among hospitalized patients*.* Due to common use of broad-spectrum antibiotics, aging of the population, and an increasing number of severe diseases requiring healthcare interventions, problem of CDI is growing [4–8]*.* The rate of CDI has doubled over the last 10 years [9, 10]. There is a high rate of asymptomatic colonization (14–70% in infants, 6% in children < 2 years of age) described in healthy population [3]. *C*. *difficile* strains usually produce two toxins: cytotoxin B and enterotoxin—toxin A. Sometimes binary toxin can be present, which is connected with severe CDI course. The main symptoms of infection are diarrhea, vomiting, abdominal pain, and fever [11–13]. Sometimes, toxic megacolon or sepsis can occur during CDI caused by more virulent serotypes. Traditional risk factors for CD-associated disease are use of broad-spectrum antibiotic, H2 blockers, chemotherapy, prolonged immunosuppression, and long-term hospitalization [14, 15].

CD colitis is a serious complication in immunosuppressed patients which can even end up in septicemia and a fatal outcome [16–19]. This population needs a special attention due to a higher incidence of the disease, presenting greater difficulty in the interpretation of diagnostic tests because of possibility of other causes of diarrhea. One of these groups is allogeneic hematopoietic stem cell transplant (HSCT) recipients who remain at high risk for CDI, with incidence ranging from 2 to 27% [14, 15]. Numerous risk factors including acute graft-versus-host disease (aGVHD), HLA mismatch status, intensity of conditioning, or the use of total body irradiation (TBI) in conditioning regimens may play an important role in the course of CDI in these patients [15, 20–23]. The aim of this retrospective multi-center nationwide study was to determine the incidence, clinical course, and outcome of CDI in pediatric patients after HSCT or receiving standard chemotherapy.

## Materials and methods

### Patients

Between 1 January 2012 and 31 December 2015, a total number of 1846 pediatric patients were treated for malignancy and 342 HSCTs were performed in 15 Polish oncological centers. Children were divided into two groups: patients treated with standard chemotherapy and/or radiotherapy for hematological malignancies or solid tumors (PHO group) and transplant recipients (HSCT group). Informed consent was obtained from parents of participants of the study.

Patients in PHO and HSCT groups were divided into three subgroups according to primary diagnosis: group C—acute lymphoblastic leukemia (ALL), acute myeloblastic leukemia (AML), Hodgkin lymphoma (HL), non-Hodgkin lymphoma (NHL), and myelodysplastic syndromes (MDS); group L—solid tumors (ST); and group B—other (non-malignant diseases, in HSCT group only).

### Prophylaxis of infections

In PHO group, prophylactic trimethoprim/sulfamethoxazole was administered in all patients starting from the day of diagnosis of malignancy. This prophylaxis was suspended during treatment with methotrexate in ALL patients and during high doses of cytarabine in AML patients. Patients with acute leukemia received mold-active prophylaxis with itraconazole or posaconazole. In the remaining low-risk cohorts, oral fluconazole prophylaxis was used.

All HSCT patients were followed starting from day of transplantation up to at least 6 months post transplantation. HSCT patients were hospitalized in single-bed rooms. Antimicrobial prophylaxis consisted of oral ciprofloxacin and colistin from the beginning of the conditioning regimen. Oral or intravenous acyclovir and oral posaconazole were started at the onset of allogenic conditioning regimen, while fluconazole was used in autologous transplantation. Prophylactic trimethoprim/sulfamethoxazole was given to all patients before and after HSCT until at least 1 month after the end of immunosuppression. Antifungal prophylaxis with posaconazole was continued until the end of immunosuppressive therapy. First-line intravenous antibiotic empiric therapy usually included a broad-spectrum beta-lactam and aminoglycoside.

### Diagnosis of CDI

CDI was diagnosed in patients having diarrhea (more than three semiliquid or liquid stools within 24 h) and a positive stool test result for the presence of toxigenic *C*. *difficile* (as detected by enzyme immunoassay, polymerase chain reaction, or microbial culture), without evidence of another infectious cause (rotavirus, adenovirus, *Salmonella*, *Shigella*, *Cryptosporidium*) [3, 14]. Stool specimens were tested each time in all patients having diarrhea. Additionally, asymptomatic HSCT patients were weekly screened for the presence of *C*. *difficile*, rotavirus, adenovirus, and multi-resistant bacteria, as a part of routine monitoring. Ribotyping and endoscopic examination was not routinely performed; gastroscopy and/or colonoscopy was done in case of suspicion of gut GVHD.

Time to CDI diagnosis in PHO group was defined as a time from the day of beginning of oncological therapy to CDI episode, while in HSCT patients from the day of transplant to CDI episode.

CDI recurrence was defined as a new episode of clinically and microbiologically documented CDI separated by complete resolution of clinical symptoms of primary infection with at least 4 weeks interval.

Severe CDI course was defined by CDI first-line treatment failure which meant lack of improvement in stool consistency after 3 days, the need to add the second drug, new signs of severe colitis, the need of admission to an intensive care unit, and/or death related to CDI [17, 18].

### Treatment of CDI

In case of clinical symptoms and positive A or B toxin or binary toxin of CD before high-dose chemotherapy, treatment with oral metronidazole (3 × 10 mg/kg) was used for 10–14 days. In case of busulfan-containing regimens, oral vancomycin (4 × 10 mg/kg) was usually administered. In case of severe clinical course of CDI, intravenous metronidazole, vancomycin, teicoplanin (10 mg/kg every 12 h for three doses, then once daily), or oral rifaximin (3 × 200 mg) were administered, usually in combination therapy.

### Statistical analysis

To compare differences between groups, the chi-square test or Fisher exact test was used for categorical variables and the Mann-Whitney *U* test for continuous variables. Hazard ratio (HR) and 95% confidence intervals (95%CI) are shown. The cumulative incidences of CDI were assessed using competing risk analysis and Gray’s test. A multivariate logistic regression using the stepwise model selection method was used to evaluate potential risk factors that might influence donor outcome variables. The following risk factors were analyzed in PHO group: age, primary diagnosis, time from diagnosis to CDI, and duration of treatment. The following risk factors were analyzed in HSCT group: age, primary diagnosis, time from diagnosis to CDI, duration of treatment, type of transplant (allo vs auto), HLA match, intensity of conditioning (myeloablative vs others), the use of TBI, source of graft (bone marrow vs peripheral blood), and GVHD. *P* < 0.05 was regarded as significant.

## Results

### Demographics

PHO group (*n* = 1846) included 386 children with ALL, 74 with AML, 114 with NHL, 108 with HL, and 1105 with ST, and 59 were diagnosed with other diseases. HSCT group included 342 transplants (267 allogenic and 75 autologous). Children were transplanted due to ALL (*n* = 86), AML (*n* = 51), NHL/HL (*n* = 23), severe aplastic anemia (SAA) and other bone marrow failure syndromes (*n* = 53), ST (*n* = 62), PID (*n* = 36), or other diseases (*n* = 31).

### Incidence of CDI

**Fig. 1:**
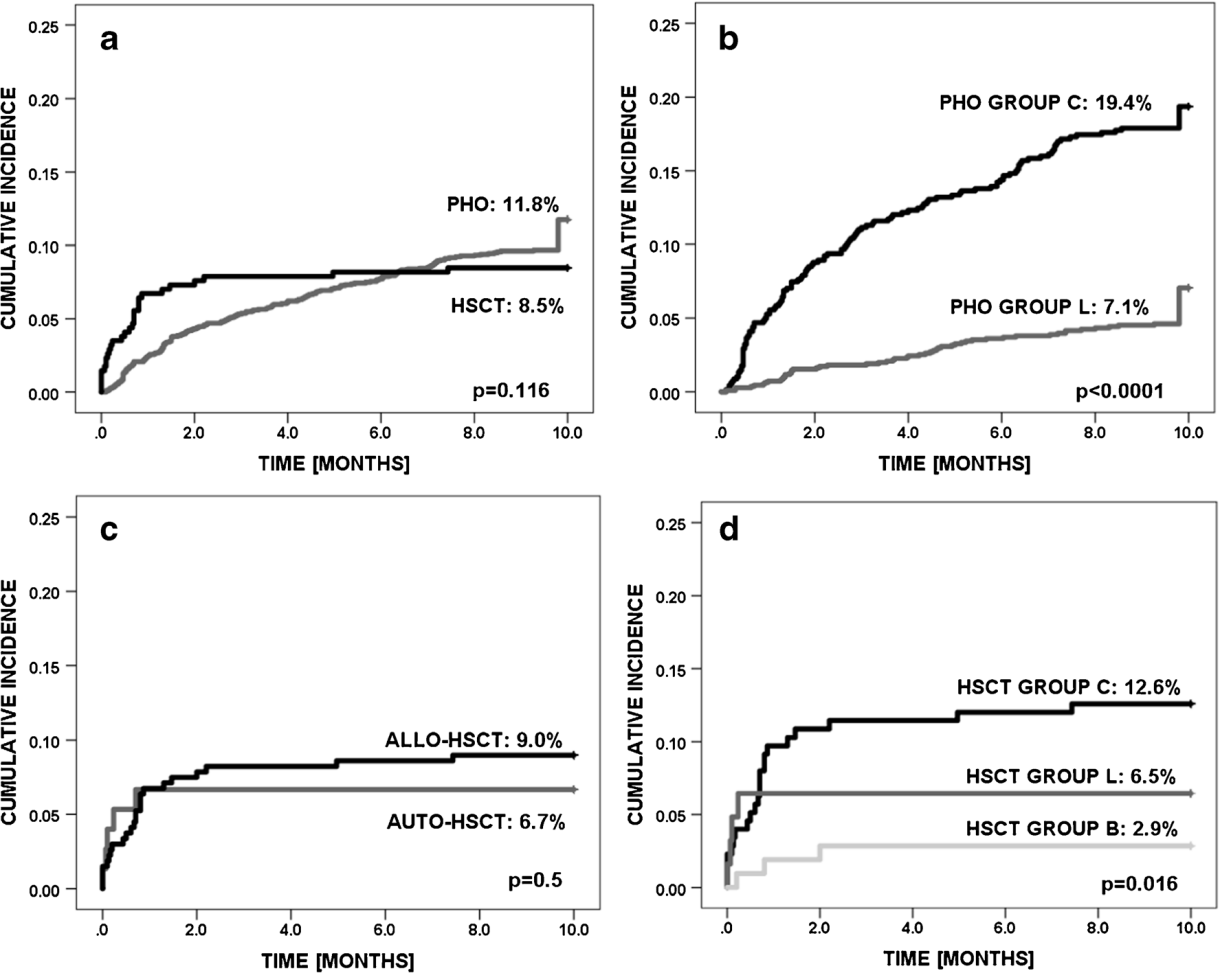
Cumulative incidence of infection with *Clostridium difficile* in: (**a**) PHO vs HSCT patients; (**b**) PHO patients group C vs group L; (**c**) allo-HSCT vs auto-HSCT patients; (**d**) HSCT patients group C vs group L vs group B. CDI occurring after 10 months are shown on the graphs at that time point

The overall time-dependent cumulative incidence of CDI was comparable in PHO and HSCT patients (11.8 ± 0.8% vs 8.5 ± 1.5%) (Fig. 1a). In PHO group, CDI were observed in 210 (14%) patients, including 106 boys and 104 girls (median age 6.5 years; range 0.12–18). The incidence of CDI in PHO group reached 21% for ALL, 19% for AML, 17% for NHL, and 11% for HL (Table 1). In ST group, the highest incidence occurred in Ewing sarcoma (ES; 20%), followed by neuroblastoma (NBL; 9%), rhabdomyosarcoma (RMS; 8%), and Wilms tumor (WT; 6%). The risk of CDI was higher in group C than in group L (18.5 vs 7.6%; HR = 2.8, 95%CI = 2.0–3.7; *P* < 0.0001) (Fig. 1b).

**Table 1:** Frequency of CDI in PHO and HSCT patients with respect to primary diagnosis

	PHO	HSCT	HR (95%CI)	*P*
Acute lymphoblastic leukemia	81/386 (21.0%)	12/86 (13.9%)	1.6 (0.8–3.1)	0.13
Acute myeloblastic leukemia	14/74 (18.9%)	6/51 (11.8%)	1.7 (0.6–4.9)	0.28
Non-Hodgkin lymphoma	19/114 (16.7%)	2/13 (15.4%)	1.1 (0.2–5.5)	0.90
Hodgkin lymphoma	12/108 (11.1%)	1/10 (10.0%)	1.1 (0.2–9.6)	0.91
Solid tumors	84/1105 (7.6%)	4/62 (6.5%)	1.2 (0.4–3.3)	0.74

In HSCT group, episodes of CDI were diagnosed in 29 (8%) patients, including 24 (8.9%) in allo-HSCT recipients and 5 (6.7%) in children after auto-HSCT (Fig. 1c). Twenty-one out of 29 children were diagnosed with hematological malignancies. The median age of patients was 9.3 years (range 2.5–19.0). The incidence was 14% for ALL, 12% for AML, 13% for lymphomas, 6% for MDS, and 5% for SAA. The highest incidence among ST patients was 8% for ES and 6% for NBL. The incidence of CDI was highest in group C in comparison to that in group L and group B (Fig. 1d). The incidence was higher in group C vs that in other patients (HR = 3.3; 95%CI = 1.4–7.9; *P* = 0.005), as well as in patients with malignant diseases vs non-malignant ones (4.2; 95%CI = 1.2–14; *P* = 0.01). Majority (24/29) of infected patients underwent myeloablative conditioning. In allogeneic setting, 21/24 patients underwent matched unrelated donor (MUD-HSCT), 2/24 had matched sibling donor (MSD-HSCT), and one patient was given a haploidentical transplantation. Bone marrow and peripheral blood were the transplant source in 7/24 and 17/24 allo-HSCT patients with CDI, respectively.

### Clinical course/manifestation of CDI

The main symptoms in PHO group were diarrhea and fever. The median time to develop CDI in all groups was 6.3 months from the diagnosis of the malignancy and beginning of anticancer therapy. CDI occurred earlier in group C than in group L patients (2.6 vs 5.5 months; *P* < 0.001).

The median time to the CDI was shorter in AML patients than in ALL patients (1.9 vs 3.4 months, *P* = 0.059). Median time of CDI treatment was 12 days (range 7–203 days). Anti-CDI treatment was successful in first-line therapy with metronidazole (138/159; 86.8%), vancomycin or teicoplanin (28/28; 100%), and rifaximin (4/5; 80%). The success rate in second-line treatment was 100% for vancomycin (13/13), rifaximin (8/8), and metronidazole (1/1). Nine patients were not given any medications, and data were not available in nine patients. Several patients were given intravenous immunoglobulins. Probiotics were used rarely due to neutropenia, and no fecal microbiota transplantation (FMT) was performed among PHO patients during the period of the study.

In patients after HSCT, the median time to develop CDI was 21 days (range 2–180 days) after HSCT. CDI occurred in 82.8% of cases during the first month after transplantation. The median time of treatment of CDI among HSCT recipients was 12 days (range 7–98 days), and it was the same as in PHO group. Treatment was successful in first-line therapy with metronidazole (13/24; 54.2%), vancomycin (1/2), and rifaximin (2/3). The second-line treatment was successful in 92.3% cases after the use of vancomycin (6/7) and rifaximin (5/5) or metronidazole (1/1). In HSCT group, probiotics were used rarely and FMT was not performed during the period of the study. In case of hipogammaglobulinemia (< 5 g/L), the majority of patients received immunoglobulin supplementation.

The results of first-line treatment of CDI were better in PHO vs those in HSCT patients (88.5 vs 55.2%; HR = 6.3; 95%CI = 2.6–14; *P* < 0.0001). The efficacy of metronidazole in first-line treatment of CDI was higher in PHO vs that in HSCT (86.8 vs 54.2%; HR = 5.5; 95%CI = 2.2–14; *P* < 0.0001).

Clinically and microbiologically documented CDI recurrences were observed in 31/210 (14.8%) PHO and in 6/29 (20.7%) HSCT patients (HR = 0.6; 95%CI = 0.3–1.7; *P* = 0.8). One case of death with concomitant CDI was reported, in 17-year-old boy with SAA (MUD, HLA 10/10) with no signs of aGVHD. The primary cause of death in these cases was a generalized inflammatory response syndrome with multi-organ failure.

### Multivariate analysis for clinical severity of CDI

In multivariate analysis (Table 2) in PHO group, patients under 5 years had milder course of disease (HR = 1.8; *P* = 0.048). Patients with diagnosis of acute leukemia had clinically more severe disease (HR = 3.1; *P* = 0.036). There was no significant impact of sex, time to CDI, and duration of treatment. Among children who underwent HSCT, no factor contributed to severity of disease and outcome, although there was a trend towards more severe clinical course of CDI in patients treated for acute leukemia or older than 5 years (Table 2).

**Table 2:** Multivariate logistic regression analysis for risk factors of severe clinical course of CDI

	PHO*		HSCT*	
	HR (95%CI)	*P*	HR (95%CI)	*P*
Diagnosis of acute leukemia	3.1 (1.1–8.6)	0.036	1.4 (0.98–2.1)	0.076
Age > 5 years	1.8 (1.0–5.2)	0.048	1.6 (0.8–6.3)	0.097

*All other factors were not significant

## Discussion

Patients at high risk of CDI include solid organ or hematopoietic transplant recipients. The incidence of CDI in HSCT group varies between 2 and 27% [14, 15]. Some variations between studies might depend on cohort size and percentage of autologous recipients [24]. Patients after HSCT had CDI cumulative incidence of 13% at 1 year, with 50% of infections occurring during the first month after HSCT. Boyle et al. found that 11% of adults and 17% of children developed episode of CDI by day + 100 [25].

In our study, we observed incidence of 8.5% among HSCT recipients, including 8.9% allo-HSCT and 6.7% auto-HSCT. In PHO group, the incidence was 14% among all patients with malignancy. Higher rate of CDI in allogeneic HSCT recipients can occur due to a higher incidence of the main risk factors for CDI such as prolonged hospitalization, gastrointestinal mucosa damage due to chemotherapy, radiotherapy, frequent use of antibiotics, and development of gastrointestinal GVHD [26]. The type of malignancy can also influence a predisposition for CDI; however up to now, no clear relationship between the type of cancer and the risk of CDI has been described [27]. In our study, we observed a higher incidence of CDI in children with hematological malignancies both in PHO group and in patients after transplantation.

Another important risk factor is age. Among pediatric patients, the risk of CDI varies by age, as older children are regarded to have a lower rate of CDI [28–30]. Neonates and infants who are at high risk of asymptomatic carriage of *C*. *difficile* may serve as a reservoir for infection. Exposure to other children and fomites (e.g., toys) may facilitate with transmission of *C*. *difficile* and play an important role in this population.

According to our observation, severe clinical course of CDI occurred more often in older group: in PHO group, we observed that younger patients (< 5 years) had milder course of disease. Similar trend was found among HSCT recipients.

We did not find HLA matching status, intensity of conditioning, use of TBI, or GVHD to be a significant risk factor for a longer period of treatment or more severe CDI course, as some authors reported [20, 21, 23]. In a case-control study of 37 allogeneic HSCT recipients with CDI, Dubberke et al. [31] found an increased risk of GVHD, severe GVHD, and gut GVHD after CDI. In multivariate analysis, they observed that aGVHD and the use of cord blood as the source of stem cells were identified as risk factors for CDI. Chakrabarti et al. [20] also described aGVHD grades 3 to 4 to be a risk factor for CDI, and that study was the first to report the use of cord blood as a risk factor for CDI. Relationship between CDI and GVHD still remains a complex issue. CDI may increase the risk of GVHD during the first year after infection, what can suggest that microbial antigenicity and/or the patient response to the infection may contribute to the pathogenesis of GVHD [24]. Boyle et al. found that overall gastrointestinal GVHD significantly predicted the development of CDI between 2 months and 1 year after transplant [25].

The main manifestations of CDI infection are diarrhea and abdominal pain which can also be found in patients with other infections such as adenovirus, norovirus, *Salmonella*, *E*. *coli*, norovirus, and colitis ulcerosa and in patients suffering from inflammatory diseases or receiving anti-inflammatory (e.g., steroids) or antiviral drugs [14, 32]. Chakrabarti et al. found that frequent co-infections which cause abdominal pain, fever, or diarrhea may mislead to an overestimation of the rate of CDI. On the other hand, it is also true that patients with asymptomatic colonization can progress to symptomatic infection [20].

Treatment algorithm for CDI in hematological patients depends on the severity of infection. Metronidazole remains still the “gold standard” in treatment of CDI. Due to the intolerance or in case of recurrent infection (frequent presence of metallic taste, mucositis, and nausea in patients treated with metronidazole), some authors recommend to use vancomycin. For patients with complicated CDI with ileus or toxic colitis and/or significant abdominal distention, vancomycin delivered orally (500 mg four times per day) and/or per rectum (500 mg in a volume of 500 mL four times a day) plus intravenous metronidazole (500 mg three times a day) is the treatment of choice. Supportive care including intravenous fluid resuscitation and electrolyte replacement should be delivered to all patients with severe CDI. In the absence of significant abdominal distention or ileus, oral or enteral feeding should be continued [33, 34]. In case of neutropenia and possibility of sepsis in immunocompromised patients, probiotics should be used with caution [35, 36]. Nowadays, new antibiotics including surotomycin, cadazolid, and ridinilazol have been developed for the treatment of CDI. Recent clinical studies demonstrated bezlotoxumab (anti-CD toxin B monoclonal antibodies) to be a valuable therapeutic option [37, 38].

Metronidazole was the first choice in our study population. In severe cases, we used vancomycin in monotherapy, or together with metronidazole in combination therapy. In some cases, we administered also rifaximin or teicoplanin with very good efficacy. The clinical presentation of CDI in pediatric patients in most cases presented as mild-to-moderate diarrhea, abdominal pain, and vomiting. Severe complications of CDI, such as fulminant CDI or pseudomembranous colitis, are not common among children [16–18]. Neither Antonara et al. [17] nor Chakrabarti et al. observed severe CDI or death attributing to CDI [20]. Contrary, in the other study of 37 patients, the increased risk of death was evaluated at 180 days and restricted to patients with severe CDI (57% of patients) [39]. Raising incidence of CDI associated with the emergence of more virulent strains since 2002 raises new concerns regarding the impact on mortality, especially in immunocompromised patients [20]. In our study, most of patients experienced relatively mild CDI with good response to antibiotic therapy.

## Conclusions

CDI occurred in 14% PHO and 8.5% HSCT patients, including 6.7% patients after autologous HSCT. The highest incidence occurred in patients with hematological malignancies both after transplantation and during conventional chemotherapy. Children with AML have shorter time to CDI than patients with ALL; however, most of patients experienced relatively mild clinical course with adequate response to antibiotic therapy. We confirm metronidazole to be the first choice option in CDI treatment in PHO patients. Almost all children responded well to the treatment with limited adverse effects, observed only in a few patients. CDI rarely contributed to death among children during conventional oncological treatment or in transplant recipients. In non-HSCT children, younger age (< 5 years) and diagnosis other than acute leukemia were the prognostic factors for less severe clinical CDI course.
